# Economic Status, Education and Empowerment: Implications for Maternal Health Service Utilization in Developing Countries

**DOI:** 10.1371/journal.pone.0011190

**Published:** 2010-06-23

**Authors:** Saifuddin Ahmed, Andreea A. Creanga, Duff G. Gillespie, Amy O. Tsui

**Affiliations:** Population, Family and Reproductive Health Department, Johns Hopkins Bloomberg School of Public Health, Baltimore, Maryland, United States of America; CIET, Canada

## Abstract

**Background:**

Relative to the attention given to improving the quality of and access to maternal health services, the influence of women's socio-economic situation on maternal health care use has received scant attention. The objective of this paper is to examine the relationship between women's economic, educational and empowerment status, introduced as the *3Es*, and maternal health service utilization in developing countries.

**Methods/Principal Findings:**

The analysis uses data from the most recent Demographic and Health Surveys conducted in 31 countries for which data on all the *3Es* are available. Separate logistic regression models are fitted for modern contraceptive use, antenatal care and skilled birth attendance in relation to the three covariates of interest: economic, education and empowerment status, additionally controlling for women's age and residence. We use meta-analysis techniques to combine and summarize results from multiple countries. The *3Es* are significantly associated with utilization of maternal health services. The odds of having a skilled attendant at delivery for women in the poorest wealth quintile are 94% lower than that for women in the highest wealth quintile and almost 5 times higher for women with complete primary education relative to those less educated. The likelihood of using modern contraception and attending four or more antenatal care visits are 2.01 and 2.89 times, respectively, higher for women with complete primary education than for those less educated. Women with the highest empowerment score are between 1.31 and 1.82 times more likely than those with a null empowerment score to use modern contraception, attend four or more antenatal care visits and have a skilled attendant at birth.

**Conclusions/Significance:**

Efforts to expand maternal health service utilization can be accelerated by parallel investments in programs aimed at poverty eradication (MDG 1), universal primary education (MDG 2), and women's empowerment (MDG 3).

## Introduction

The disparity in maternal mortality between poor and rich regions of the world is striking. In 2005, the maternal mortality ratio was highest in developing regions (450 maternal deaths per 100,000 live births), in severe contrast to developed regions (9 maternal deaths per 100,000 live births) and countries of the commonwealth of independent states (51 maternal deaths per 100,000 live births) [1]. At the global level, maternal mortality has decreased at an average of less than 1% annually between 1990 and 2005, far below the 5.5% annual decline necessary to reach the 5th Millennium Development Goal (MDG), that is to reduce maternal mortality by three-quarters by 2015 [1]. Numerous authors suggest that the vast majority of maternal deaths are preventable by giving women access to relatively basic maternal health services [2]–[7]. Conditions amenable to intervention by skilled health providers are involved in about 80% of maternal deaths, and thus, to date, the core strategy for driving down maternal mortality has been to increase access to emergency care around the time of delivery [2]. While skilled birth attendance and emergency obstetric care are essential to securing significant reductions in maternal mortality, health service expansion by itself is unlikely to be enough.

Underutilization of available maternal health services has been found in areas where the need for such services is greatest [8]–[11], i.e., among disadvantaged populations. The latter are less healthy and in more need of health services than their better-off counterparts. Decades ago, Hart posited his *law of inverse care* wherein those least in need of health care–the healthy and wealthy–are more likely to receive care than the sick and the poor [12]. The saliency of Hart's law is currently well recognized and documented [13], [14]. Moreover, women from disadvantaged segments of society are in double jeopardy since they find themselves even more disadvantaged within a disadvantaged population–they will be the poorest among the poor and the least educated among the inadequately educated.

Relative to the attention given to improving the quality of and access to health services, the influence of women's socio-economic situation on maternal health has received less attention. Within the health community, there is concern that health intervention programs are often solely supply-oriented and ignore the social factors constraining the demand for, access to and effective use of health services. Studies have consistently shown that women's educational attainment, social status, household wealth and decision-making power are associated with care-seeking behaviors for maternal health services and maternal survival [14]–[25]. A recent study by Kruk et al. [26] finds that higher levels of health expenditure do not automatically mean substantially greater use of skilled birth attendants by poor women; also, they find that at any given level of health care spending poor women's use of skilled birth attendants varies substantially, depending on equity in the distribution of education. Thus, a comprehensive approach to increasing health service utilization should give attention to the demand, as well as supply, side of health care delivery.

This analysis investigates inequities in use of maternal health services and estimates the magnitude of the relationships between women's economic, educational and empowerment status (introduced here as the *3Es*) and the utilization of maternal health services. The *3Es* are specifically linked to the achievement of MDGs 1 (eliminating extreme poverty), 2 (promoting universal primary education) and 3 (promoting gender equality and women's empowerment). We examine the *3Es'* role in inequitable use of three key service components of maternal health programs: pre-conceptional care (use of modern contraception), gestational care (antenatal care) and delivery care (skilled attendance at birth) in 33 low-income countries. Inequitable use exists when an undesirable health situation is disproportionately found among social and economic groups despite known effective interventions being available [27]. Inequities in service utilization as a result of economic, educational and empowerment differentials do not carry the same gravity and ethical implications as inequities in preventing deaths. However, if such inequities are negatively associated with access to and use of life-saving maternal health services, they are functionally and morally equivalent to inequities in mortality prevention.

Thaddeus and Maine identified three groups of factors obstructing access to care in their *3Ds* model–delay in seeking care, delay in arriving at a health facility, and delay in the provision of care [28]. Neither the *3Ds* nor the currently described *3Es* incorporate all influences on the use of maternal health services, much less maternal mortality. Nonetheless, one can conceptualize the *3Ds* as proximate conditions of maternal morbidity and mortality, and the proposed *3Es* as antecedents to the *3Ds.* For example, lack of education can prevent awareness of life-threatening obstetrical complications, which in turn reduces women's recognition of the need to seek risk-appropriate health care. Women's limited decision-making power, as well as constrained economic resources, likewise can inhibit their ability to seek health services and/or contribute to delays in accessing and receiving medical care even in places where services are readily available. Moreover, economic inequities may lead to substandard care offered to impoverished women who ultimately have the highest likelihood of dying during pregnancy, delivery or immediately post-partum.

While the direction of the associations between the *3Es* and women's use of health services is well known, the magnitude of these associations is not. Women's economic, educational and empowerment status are each related to one of the first three MDGs. In order to inform health policies and advocacy efforts aimed at achieving the MDGs by 2015, it is of utmost importance to know how much changes in women's economic, educational and empowerment status can contribute to increase women's utilization of health care services.

## Methods

Our analyses examine the relationships between the *3Es* and maternal health care utilization, specifically use of modern contraception, attendance of four or more antenatal care visits as per the WHO recommendation [29], and skilled birth attendance. We use Demographic and Health Survey (DHS) data from 33 countries for which all variables of interest are available [30]. DHSs are nationally representative surveys that employ standardized questionnaires to collect extensive data from women of reproductive age (15 to 49 years) in developing countries. The DHS obtains information on women's socio-demographic characteristics, their reproductive behaviors, birth history and maternal health service utilization. We use data on all births to women in the five years prior to the survey year to examine women's experiences with antenatal care and skilled birth attendance, and data on modern contraceptive use among all the women interviewed for the country-specific DHSs.

We define our *3Es* measures to reflect achievement of MDGs 1–3. The DHS lacks questions on household income and consumption expenditures, but a widely employed asset or wealth index is available based on household ownership of various assets and on housing characteristics [31]. The index is generated through a principal-components analysis to score household wealth assets. Subsequently, the score distribution is divided into quintiles, from the poorest 20% to the richest 20%, and women are assigned their household's quintile classification. MDG 1 is to “eliminate extreme poverty” and therefore, we compare women in the poorest with those in the richest wealth quintile. For women's education, in order to assess disparities that specifically relate to MDG 2, i.e., to achieve universal primary education, we compare women with complete primary education to those with no or incomplete primary education. For MDG 3, i.e., to promote gender equality and empower women, we use a composite score based on the set of women's autonomy questions specifically asked in the DHS. This variable is the sum of positive (“yes”) answers given to five distinct questions about women's involvement in decisions related to their own health care, large household purchases, daily household needs purchases, visits to their family or relatives and daily meal preparation. This sum can range from 0 to 5, and in our analyses, we compare women with an empowerment score of 5 to those with a score of 0.

We fit logistic regression models for the following three service utilization outcomes: 1) modern contraceptive use, 2) attendance of four or more antenatal care visits as recommended by the WHO, and 3) skilled attendance at birth. In addition to the *3Es*, each model adjusts for the woman's age (years) and residence (urban/rural). The estimated standard errors of the log odds ratios are adjusted as well for the complex survey design using the Taylor linearization method [32].

We use meta-analytic techniques to combine and summarize the results from multiple countries. Considering the heterogeneity among the countries, we fit random effects models using the DerSimonian and Laird method [33]. This method is extensively used in the literature for meta-analyses of randomized controlled trials, cohort, case-control, as well as cross-sectional studies such as DHSs. The pooled odds ratios estimate the average weighted association between the three health service utilization outcomes and each of the *3Es*. We investigate the quantitative and qualitative heterogeneity among the 33 country surveys included, as well as the influence of single surveys on the overall meta-analysis estimate through sensitivity analyses. Exploratory analyses showed that primary education is almost universal in Moldova and Armenia, and therefore, country-specific estimates for the associations between education and maternal health care use cannot be obtained for these two countries. Analyses conducted on all 33 countries and after excluding Armenia and Moldova showed that the pooled estimates do not change significantly; thus, for consistency, we present the results after excluding these two countries from all the pooled analyses.

All analyses are performed using Stata version 9.1 (Stata Corporation, College Station, TX).

## Results

**Figure 1:**
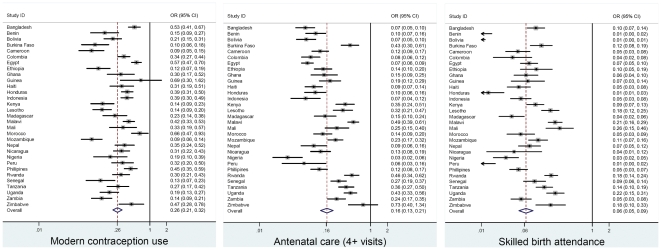
Meta-analysis of the regression model results of maternal health care utilization on women's economic status: 31 developing countries. *Note:* Comparing women in the poorest with women in the richest wealth quintile.

**Figure 2:**
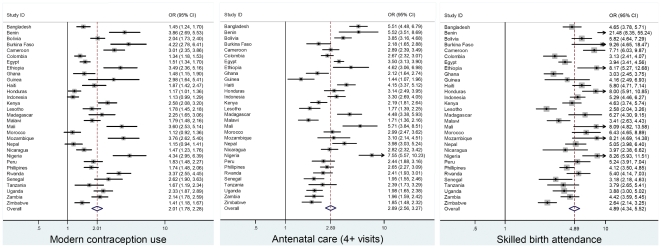
Meta-analysis of the regression model results of maternal health care utilization on women's education: 31 developing countries. *Note:* Comparing women with complete primary education with women with no or incomplete primary education.

As expected, women experiencing inequities in the *3Es* in the 31 countries (21 of which are in Africa) are less likely to use health services than women who are better-off. Table 1 summarizes the meta-analysis results, pooled adjusted odds ratios (ORs) and their confidence intervals (CI), for maternal health service use among women in the 31 countries and, as a sub-analysis, for women in African countries. Results for the 31 countries closely match those for the 21 African countries suggesting the importance of the *3Es* for women everywhere. As seen in Figure 1, the odds of the poorest 20% compared to the richest 20% of women are 74%, 84% and 94% lower for use of modern contraception, attendance of four or more antenatal care visits and skilled attendance at delivery, respectively (pooled-OR: 0.26, 95% CI 0.21–0.32; pooled-OR: 0.16, 95% CI 0.13–0.21; and, pooled-OR: 0.06, 95% CI 0.05–0.09). The most dramatic pattern of association observed is for inequities in education (Figure 2). Women who have completed primary education are almost five times more likely to have had a skilled birth attendant at delivery (pooled-OR: 4.89, 95% CI 4.34–5.52) than less educated women. Furthermore, women with complete primary education are almost three times more likely to have made at least four antenatal care visits (pooled-OR: 2.89, 95% CI 2.56–3.27) and twice as likely to use modern contraception (pooled-OR: 2.01, 95% CI 1.78–2.28) than women with no or less than primary education (Figure 2). The pattern for the subsample of African countries is very similar.

**Table 1:** Pooled odds ratios and 95% confidence intervals from meta-analysis of maternal health care service utilization on women's economic, educational and empowerment status: 31 developing countries.

	Millennium Development Goals and the *3Es*		
	MDG 1: Eliminate extreme poverty	MDG 2: Achieve universal primary education	MDG 3: Empower women
Use of maternal health care services	Economic status^*^ pooled OR (95% CI)	Educational status^**^ pooled OR (95% CI)	Empowerment status^***^ pooled OR (95% CI)
Modern contraception			
All countries	0.26 (0.21, 0.32)	2.01 (1.78, 2.28)	1.82 (1.52, 2.17)
African countries	0.23 (0.17, 0.31)	2.40 (2.01, 2.87)	1.49 (1.28, 1.73)
Antenatal care (4+ visits)			
All countries	0.16 (0.13, 0.21)	2.89 (2.56, 3.27)	1.52 (1.37, 1.66)
African countries	0.22 (0.16, 0.30)	2.74 (2.31, 3.25)	1.29 (1.18, 1.40)
Skilled birth attendance			
All countries	0.06 (0.05, 0.09)	4.89 (4.34, 5.52)	1.31 (1.11, 1.54)
African countries	0.09 (0.07, 0.12)	4.81 (4.07, 5.70)	1.19 (1.06, 1.33)

*Note:* All figures are statistically significant at a level p<0.05; ^*^ Comparing women in the poorest with women in the richest wealth quintile; ^**^ Comparing women with complete primary education with women with no or incomplete primary education; ^***^ Comparing women with the highest to women with no decision-making power.

**Figure 3:**
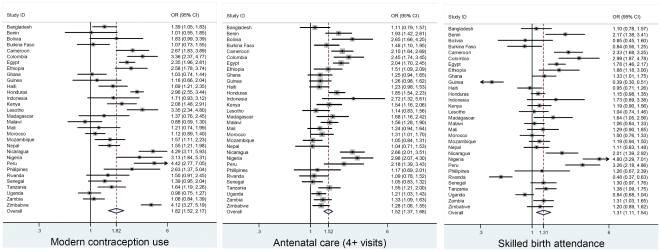
Meta-analysis of the regression model results of maternal health care utilization on women's empowerment: 31 developing countries. *Note:* Comparing women with the highest to women with no decision-making.

Similarly, inequities in women's empowerment in the 31 countries are associated with lower maternal health service utilization, although not as substantially as for the other 2Es. The most pronounced association for women's empowerment is with modern contraceptive use. Women with the highest empowerment score (5) have an 82% higher odds of using modern contraception than women with a zero empowerment score (pooled-OR: 1.82, 95% CI 1.52–2.17). Similarly, women with the maximum as compared to the minimum empowerment score are, respectively, 1.52 (95% CI 1.37–1.66) and 1.31 (95% CI 1.11–1.54) times significantly more likely to have attended four or more antenatal care visits and have had a skilled birth attendant (Figure 3).

## Discussion

This analysis deepens our awareness of the wide ranging, strong and persistent associations of three key socio-economic factors with utilization of critical services that influence maternal health in 31 developing countries with approximately one fifth (18.9%) of the world's population. The paper introduces the *3Es* (women's economic, educational and empowerment status) and shows their direct linkage to the uptake of three of the most basic maternal health services. While studies conducted in various individual countries have consistently shown that women's household wealth, educational attainment and decision-making power are associated with the use of maternal health services and maternal survival, this study is the first to provide multi-country evidence from the developing world of the magnitude of their association with modern contraception use, antenatal care and skilled birth attendance. Of the three socio-economic factors under study, women's empowerment is the least strong factor associated with women's use of maternal health services in all countries, and especially so among African countries. Not many studies have extensively investigated the association between women's empowerment and the use of one or more reproductive health services. Most recently, Fotso et al. have identified women's household wealth and education as strong correlates for place of delivery in Nairobi, Kenya, while the association with women's autonomy was weaker [34].

While our analysis supports Hart's *inverse care law*–that the more disadvantaged a population the less likely they are to have accessible health services–neither the poor nor the rich can use services that do not exist. Efforts to lower maternal mortality will fail in the absence of basic maternal health services, which, in turn, are unlikely to become available without pro-poor health policies [35]. In places where health services are available, they often fall short of being patient-friendly. It is reasonable to assume that an expansion of high quality services may increase women's motivation to use these services [36]. If so, women with means, in terms of education, decision-making autonomy and access to economic resources, are more likely to cope effectively with the challenges presented by health systems.

This analysis is not without limitations. Importantly, the analyses draw on cross-sectional data; and thus, only associations and no causal relationships are examined. The pooled cross-country analyses employ random effects models to account for the *quantitative* heterogeneity among the countries included in the analysis. However, our inability to control all sources of *qualitative* heterogeneity among data from 31 countries might have biased our estimates of the relationship between the *3Es* and maternal health service use. While the DHSs offer the unique advantage of performing cross-country analyses of data collected using a standardized questionnaire and methodology, when interpreting these results one needs to consider that surveys included in this analysis were conducted over an 8-year period (1998–2006) and represent populations in a select and limited number of developing countries.

The empowerment variable used in our analyses is not a validated measure of women's decision-making power. There is no current scientific consensus on the construct of women's empowerment; thus, we conducted preliminary analyses testing three different such measures: (1) the empowerment score described above, (2) the one of five women's decision making variables in the DHS that had the most pronounced relationship with the study outcomes (i.e. women's autonomy to decide on their own health), and (3) the gender equity index developed by Social Watch in 2005 [37]. Use of all three measures produced similar results. We present the findings using the empowerment score in order to make use of all available data in the context of qualitative heterogeneity (i.e. cultural diversity) among the countries studied. The considerable variation between countries with regard to the relationship between women's empowerment and their use of maternal health services draws attention to the need to develop locally sensitive and meaningful measures of women's empowerment. It is clear that a globally standardized measure cannot adequately reflect differences between individual cultures and the specific needs of women in various countries. Despite measurement limitations, this analysis clearly demonstrates a significant and positive relationship between women's empowerment and maternal health service utilization, and highlights the importance of enhancing women's autonomy to raise maternal health service use in developing countries.

Due to cultural differences between the countries included in this analysis, we do not consider the potential interactions between two or all the *3Es*. Future studies assessing the associations examined here in one country or in a group of culturally similar countries should consider more complex analytical approaches and include interaction terms between women's economic, educational and empowerment status as might be appropriate. More research and in-depth country-specific assessments are needed to better understand the relationships between the *3Es* and, for example, women's choice of contraceptive methods, their willingness to attend antenatal care, their preparedness to deliver in a health facility and have a skilled attendant at birth. Such research would subsequently inform the implementation of interventions and programs to better address women's health needs and to better position them to seeking and obtaining the health care they need.

There are many benefits associated with women's higher socio-economic status; among these are the reduction of infant and child mortality, better infant and child nutrition and health, lower fertility rates, enhanced participation of women in labor force and politics, protection against abuse and exploitation. Health-related policies should incorporate and address inequalities in women's education, empowerment and economic status, as improvements in the latter may yield high returns that accrue to individuals, families, and societies at large.

In conclusion, our findings suggest that substantial increase in the use of maternal health services can be achieved by accelerating socioeconomic development and effectively addressing basic human needs of schooling, economic welfare, and gender-based discrimination. Inasmuch as appropriate reproductive and obstetric services and care are essential, our study shows that there are potentially large systemic benefits to be gained with improved socioeconomic status, which will also reduce health inequities. These benefits are preventive in nature, materializing before and during pregnancy and thereby lowering the risk of maternal complications and death. Efforts to achieve the MDG 5 target of reducing maternal mortality by three-quarters between 1990 and 2015 will require not only significant investments in the expansion of appropriate maternal health services but also parallel investments in programs aimed at poverty eradication (MDG 1), universal primary education (MDG 2), and women's empowerment (MDG 3).
